# Tratamento de Pacientes com Insuficiência Cardíaca com Fração de Ejeção Reduzida (ICFER) e Fibrilação Atrial: Precisamos Falar do Quinto Pilar!

**DOI:** 10.36660/abc.20240585

**Published:** 2025-03-26

**Authors:** Mauricio Pimentel, Lucas Simonetto Faganello, Ana Paula Arbo Magalhães, Eduardo Caberlon, Leandro Ioschpe Zimerman

**Affiliations:** 1 Hospital de Clínicas de Porto Alegre Porto Alegre RS Brasil Hospital de Clínicas de Porto Alegre, Porto Alegre, RS – Brasil

**Keywords:** Insuficiência Cardíaca, Fibrilação Atrial, Ablação por Cateter

A insuficiência cardíaca com fração de ejeção reduzida (ICFER) é uma síndrome clínica complexa, caracterizada por sintomas de dispneia e de piora da capacidade funcional, resultantes da redução do débito cardíaco em pacientes que apresentam fração de ejeção do ventrículo esquerdo (FEVE) ≤40%.^1^ A ICFER é um importante problema de saúde pública, sendo uma das principais causas de internação hospitalar, apresentando elevadas morbidade e mortalidade.^2^ Baseado em grandes ensaios clínicos randomizados, o tratamento básico de pacientes com ICFER até o final da década de 90 consistia de inibidor da enzima conversora de angiotensina (IECA) ou bloqueador da angiotensina (BRA), betabloqueador e antagonista mineralocorticóide. Após cerca de duas décadas, inicia-se então uma nova era no tratamento da ICFER a partir da publicação de ensaios clínicos randomizados de novas classes de drogas: inibidores da neprisilina e receptores da angiotensina, e os inibidores do co-transportador sódio-glicose 2 (ISGLT2).^3^ A partir dessas evidências, as diretrizes passam a definir os quatro pilares do tratamento da ICFER: IECA ou inibidores da neprisilina e receptores da angiotensina, betabloqueadores, antagonista mineralocorticoide e inibidores da SGLT2.^1,4^ Além do tratamento farmacológico, o implante do cardioversor-desfibrilador e a terapia de ressincronização cardíaca também estão indicados para pacientes com determinadas características clínicas.^5^

A fibrilação atrial (FA) e a ICFER apresentam fatores de risco comuns e frequentemente coexistem, levando, consequentemente, ao agravamento do quadro clínico e à piora do prognóstico das duas condições.^6^ O diagnóstico de FA em pacientes com ICFER está associado ao aumento da mortalidade. A ICFER é um fator de risco para o aumento da incidência de FA e está associada a maior risco de acidente vascular cerebral em pacientes com FA. A importância de se buscar estabelecer qual a melhor abordagem terapêutica para pacientes com FA e ICFER levou à realização de ensaios clínicos randomizados comparando as estratégias de controle do ritmo e de controle da frequência cardíaca. O estudo *Dofetilide in Patient with Congestive Heart Failure and Left Ventricular Dysfunction* (DIAMOND-CHF) comparou o uso de dofetilide com placebo em pacientes com disfunção ventricular. Na análise dos pacientes com FA, foi demonstrado que o uso do dofetilide determinou maior taxa de reversão e de manutenção em ritmo sinusal, porém sem efeito em relação à mortalidade total.^7^ Já o estudo *Rhythm Control versus Rate Control for Atrial Fibrillation and Heart Failure* (AF-CHF) comparou as estratégias de controle do ritmo (cardioversão e droga antiarrítmica, sendo amiodarona a droga de escolha) versus controle da frequência cardíaca em pacientes com FEVE ≤ 35%.^8^ A estratégia de controle do ritmo não demonstrou redução da mortalidade cardiovascular em relação ao controle da frequência cardíaca. Importante ressaltar que durante o seguimento, 58% dos pacientes no grupo controle do ritmo apresentaram recorrência de FA. De fato, a estratégia de controle do ritmo era pouco eficaz em realmente manter os pacientes em ritmo sinusal.

A partir do estudo pivotal de Haissaguerre, a ablação por cateter da FA evoluiu e se consolidou como a alternativa terapêutica mais eficaz para a manutenção do ritmo sinusal.^9^ Ao longo dos últimos anos, os resultados da ablação são cada vez melhores e a taxa de complicações graves progressivamente menor.^10^ Ablação por cateter para tratamento de FA em pacientes com ICFER foi avaliada em ensaios clínicos randomizados que mostraram benefício em qualidade de vida, em melhora da FEVE e da capacidade funcional e redução em de mortalidade.^11-13^ Posteriormente houve a publicação do estudo Catheter Ablation for Atrial Fibrillation in Patients with End-Stage Heart Failure and Eligibility for Heart Transplantation (CASTLE-HTx), confirmando o benefício da ablação por cateter com redução da mortalidade total.^14^ Os resultados destes estudos foram avaliados recentemente em revisão sistemática e metanálise.^15^ Em análise robusta incluindo 1055 pacientes, a metanálise demonstrou que a ablação por cateter em adição à terapia médica otimizada reduziu significativamente a taxa de hospitalização por insuficiência cardíaca, de mortalidade cardiovascular e de mortalidade por todas as causas. A redução do risco relativo para mortalidade total foi de 47%. Além disso, houve melhora da FEVE e dos escores de qualidade de vida. A consistência destes resultados fundamenta a recomendação das diretrizes mais recentes que indicam a ablação por cateter de FA como estratégia para controle do ritmo em pacientes com ICFER.^16^ O benefício obtido com a ablação é ainda mais significativo nos pacientes em que a FA é a principal causa da IC (taquicardimiopatia induzida por FA).

A utilização da expressão pilares de tratamento nos documentos de diretrizes tem por objetivo reforçar a adesão a terapias com sólida demonstração de benefício em redução de morbidade e mortalidade. A figura do pilar, como algo fundamental em uma construção, busca evitar a subutilização de terapias comprovadamente eficazes no tratamento de determinada condição clínica. Considerando-se as evidências atuais e a alta prevalência de FA na IC, a estratégia de controle do ritmo, incluindo a ablação por cateter em pacientes selecionados, pode ser recomendada como o quinto pilar do tratamento. Em outra perspectiva, a ablação também precisa ser cada vez mais considerada no manejo de pacientes com FA para evitar possível deterioração futura da função ventricular. O desafio passa a ser identificar os pacientes potencialmente elegíveis e oferecer de forma universal o acesso a este tratamento dentro do nosso sistema de saúde.
