# Supplementary material for: Tratamento de Pacientes com Insuficiência Cardíaca com Fração de Ejeção Reduzida (ICFER) e Fibrilação Atrial: Precisamos Falar do Quinto Pilar!
Source: Arq Bras Cardiol. 2025 Mar 26;122(4):e20240585. [Article in Portuguese] doi: 10.36660/abc.20240585 (PMC12040384; doi:10.36660/abc.20240585)
Supplement: Translation

# Translation: Treating Patients with Heart Failure with Reduced Ejection Fraction (HFrEF) and Atrial Fibrillation: We Need to Talk About the Fifth Pillar

**DOI:** 10.36660/abc.20240585i

**Authors:** Mauricio Pimentel, Lucas Simonetto Faganello, Ana Paula Arbo Magalhães, Eduardo Caberlon, Leandro Ioschpe Zimerman

**Affiliations:** 1 Porto Alegre RS Brazil Hospital de Clínicas de Porto Alegre, Porto Alegre, RS – Brazil

**Keywords:** Heart Failure, Atrial Fibrillation, Catheter Ablation

Heart failure with reduced ejection fraction (HFrEF) is a complex clinical syndrome characterized by symptoms of dyspnea and worsening functional capacity resulting from reduced cardiac output in patients with left ventricular ejection fraction (LVEF) ≤40%.^1^ HFrEF represents an important public health problem, being one of the main causes of hospital admission, with high morbidity and mortality rates.^2^ Based on large randomized clinical trials, the standard treatment for patients with HFrEF until the late 1990s consisted of angiotensin-converting enzyme inhibitors (ACEIs) or angiotensin receptor blockers (ARBs), beta-blockers, and mineralocorticoid receptor antagonists (MRAs). After about two decades, a new era in the treatment of HFrEF began with the publication of randomized clinical trials on new classes of drugs: angiotensin receptor-neprilysin inhibitors (ARNIs) and sodium-glucose co-transporter 2 (SGLT2) inhibitors.^3^ Based on this evidence, the guidelines defined the four pillars of HFrEF treatment: ACEIs or ARNIs, beta-blockers, MRAs, and SGLT2 inhibitors.^1,4^ In addition to pharmacological treatment, the implantation of a cardioverter-defibrillator and cardiac resynchronization therapy are also indicated for patients with specific clinical features.^5^

Atrial fibrillation (AF) and HFrEF share common risk factors and often coexist, leading to exacerbation of clinical condition and poorer prognosis for both.^6^ The diagnosis of AF in patients with HFrEF is associated with increased mortality. HFrEF is a risk factor for increased incidence of AF and is associated with a higher risk of stroke in patients with AF. The importance of determining the best therapeutic approach for patients with AF and HFrEF led to randomized clinical trials comparing rhythm control and heart rate control strategies. The Dofetilide in Patients with Congestive Heart Failure and Left Ventricular Dysfunction (DIAMOND-CHF) study compared the use of dofetilide with placebo in patients with ventricular dysfunction. In the analysis of patients with AF, it was shown that the use of dofetilide resulted in a higher rate of reversion and maintenance of sinus rhythm, but with no effect on overall mortality.^7^ The Rhythm Control versus Rate Control for Atrial Fibrillation and Heart Failure (AF-CHF) study compared rhythm control strategies (cardioversion and antiarrhythmic drug, with amiodarone being the drug of choice) to heart rate control in patients with LVEF ≤ 35%.^8^ The rhythm control strategy did not show a reduction in cardiovascular mortality compared to heart rate control. It is important to note that, during follow-up, 58% of the patients in the rhythm control group had a recurrence of AF. In fact, the rhythm control strategy was not very effective in maintaining patients in sinus rhythm.

Since the pivotal study by Haissaguerre, catheter ablation of AF has evolved and become the most effective therapeutic alternative for maintaining sinus rhythm.^9^ Over the last few years, the results of ablation have continuously improved, with a progressively lower rate of serious complications.^10^ Catheter ablation for the treatment of AF in patients with HFrEF has been evaluated in randomized clinical trials, which have shown benefits in quality of life, improved LVEF and functional capacity, as well as reduced mortality.^11-13^ Subsequently, the Catheter Ablation for Atrial Fibrillation in Patients with End-Stage Heart Failure and Eligibility for Heart Transplantation (CASTLE-HTx) study was published, confirming the benefit of catheter ablation with a reduction in overall mortality.^14^ The results of these studies were recently assessed in a systematic review and meta-analysis.^15^ In a robust analysis including 1055 patients, the meta-analysis demonstrated that catheter ablation, in addition to optimized medical therapy, significantly reduced the rates of hospitalization for heart failure, cardiovascular mortality, and all-cause mortality. The relative risk reduction for overall mortality was 47%. Furthermore, there was an improvement in LVEF and quality of life scores. The consistency of these results underpins the recommendation in the most recent guidelines, which suggest catheter ablation of AF as a strategy for rhythm control in patients with HFrEF.^16^ The benefit of ablation is even more significant in patients for whom AF is the primary cause of HF (AF-induced tachycardiomyopathy).

The use of the term ‘pillars of treatment’ in the guidelines reinforces adherence to therapies with a solid demonstration of benefit in reducing morbidity and mortality. The ‘pillar’ figure, as something fundamental in a building, for example, seeks to avoid the underuse of therapies that have been proven to be effective in treating a given clinical condition. Considering the current evidence and the high prevalence of AF in HF, the rhythm control strategy, including catheter ablation in selected patients, can be recommended as the fifth pillar of treatment. From another perspective, ablation must also be more consistently considered in managing patients with AF to prevent potential future deterioration of ventricular function. Nevertheless, the key challenge lies in identifying potentially eligible patients and ensuring universal access to this treatment within our healthcare system.
